# LINE-1 Hypermethylation in Serum Cell-Free DNA of Relapsing Remitting Multiple Sclerosis Patients

**DOI:** 10.1007/s12035-017-0679-z

**Published:** 2017-07-13

**Authors:** Marina Dunaeva, Merel Derksen, Ger J. M. Pruijn

**Affiliations:** Department of Biomolecular Chemistry, Institute for Molecules and Materials and Radboud Institute for Molecular Life Sciences, Radboud University Nijmegen, PO Box 9101, NL-6500 HB Nijmegen, The Netherlands

**Keywords:** Multiple sclerosis, LINE-1, CpG, DNA methylation, Cell-free DNA

## Abstract

Concentrations of cell-free DNA (cfDNA) circulating in blood and its epigenetic variation, such as DNA methylation, may provide useful diagnostic or prognostic information. Long interspersed nuclear element-1 (LINE-1) constitutes approximately 20% of the human genome and its 5’UTR region is CpG rich. Due to its wide distribution, the methylation level of the 5’UTR of LINE-1 can serve as a surrogate marker of global genomic DNA methylation. The aim of the current study was to investigate whether the methylation status of LINE-1 elements in serum cell-free DNA differs between relapsing remitting multiple sclerosis (RRMS) patients and healthy control subjects (CTR). Serum DNA samples of 6 patients and 6 controls were subjected to bisulfite sequencing. The results showed that the methylation level varies among distinct CpG sites in the 5’UTR of LINE-1 repeats and revealed differences in the methylation state of specific sites in this element between patients and controls. The latter differences were largely due to CpG sites in the L1PA2 subfamily, which were more frequently methylated in the RRMS patients than in the CTR group, whereas such differences were not observed in the L1HS subfamily. These data were verified by quantitative PCR using material from 18 patients and 18 control subjects. The results confirmed that the methylation level of a subset of the CpG sites within the LINE-1 promoter is elevated in DNA from RRMS patients in comparison with CTR. The present data suggest that the methylation status of CpG sites of LINE repeats could be a basis for development of diagnostic or prognostic tests.

**Electronic supplementary material:**

The online version of this article (doi:10.1007/s12035-017-0679-z) contains supplementary material, which is available to authorized users.

## Introduction

Multiple sclerosis (MS) is an autoimmune disease of the central nervous system, for which the pathogenic mechanisms are only poorly understood. There are several clinical courses of MS, including relapsing remitting (RRMS), primary progressive (PPMS), secondary progressive (SPMS), and progressive relapsing (PRMS) MS [1]. RRMS is characterized by unpredictable relapses followed by periods without symptoms of disease activity (remission) and often begins with a clinically isolated syndrome (CIS) episode. MS is difficult to diagnose at early stages, as the MS signs and symptoms can be similar to those of other diseases. Currently, the process of diagnosing MS is lengthy and costly. Therefore, there is an urgent need for highly sensitive and specific diagnostic and prognostic tests, which are preferably minimally invasive. The identification of biomarkers may facilitate the development of such tests.

Cell-free DNA (cfDNA) has been detected in body fluids such as serum and plasma. Changes in the levels or fragmentation patterns of circulating cfDNA have been associated with various diseases, in particular cancer [2]. Moreover, several studies have reported that the analysis of cfDNA methylation can be useful for the early detection, diagnosis, and prognosis of different diseases [3]. In the human genome, DNA methylation occurs predominantly at CpG dinucleotides. Some CpG dinucleotides are clustered in CpG islands (CGIs). Approximately 70% of the annotated gene promoters are associated with CGIs, and the methylation of these promoter-associated CGIs is associated with transcriptional repression. Besides gene promoters, repetitive elements such as long and short interspersed nuclear elements (LINEs and SINEs) and tandem array repeats (satellite elements) contain a substantial number of CGIs. These elements comprise about 45% of the human genome and are heavily methylated in postnatal tissues to prevent their transcription [4, 5], whereas they are frequently hypomethylated in human malignancies [6–8].

LINEs are abundant, non-long terminal repeat (non-LTR) retrotransposons, which are widely but unevenly distributed in the mammalian genomes. LINE families contribute to 12% of CpG dinucleotides in the human genome [9]. The human genome contains two superfamilies of LINEs, active LINE-1 elements and extinct families of LINE-2 and LINE-3 elements [10]. Over 500,000 copies of LINE-1 are present in the human genome, of which the vast majority is 5′ truncated [11]; about 3,000copies are full length and 80–100 of these are active retrotransposons [12]. Full-length 6-kb LINE-1 retrotransposons consist of four regions: the 5′ untranslated region (UTR), which contains both sense and antisense promoters, two open reading frames: ORF1 and ORF2, which encode an RNA binding protein and a protein with reverse transcriptase and endonuclease activity, respectively, and a 3’UTR containing a polyadenylation signal (Supplementary Fig. S1) [13, 14]. A sense promoter is responsible for transcription of the LINE-1 repeats and an antisense promoter drives transcription of adjacent regions. Many LINE-1 elements show 5′ truncations and, as a consequence, are not able to propagate due to the lack of a promoter and transcription factor binding sites [15]. Both intact ORFs are also required for LINE-1 retrotransposition [16]. DNA hypermethylation in the LINE-1 promoter is important for transcriptional repression and for the inhibition of retrotransposition.

Alteration of the LINE-1 methylation status has been observed for a number of cancers [17–19], rheumatoid arthritis [20–22], and systemic lupus erythematosus [22–24]. Several studies on the pathogenesis of autoimmune diseases suggest that changes of DNA methylation at the interspersed repetitive sequences can occur under various conditions. Lymphocytes and neutrophils from patients with SLE exhibit hypomethylation of LINE-1 [24]. A similar pattern of hypomethylation of LINE-1 repeats in CD4+, CD8+ *T* cells and B lymphocytes subsets from SLE patients in comparison with healthy controls was observed [23]. A recent study on methylation of repetitive elements demonstrated hypermethylation of repetitive elements (Alu, LINE-1, and SAT-a) in whole blood of MS patients compared to healthy controls [25]. Methylation levels of LINE-1 and Alu were correlated with EDSS scores.

We have previously demonstrated that there is no difference in cfDNA levels between patients with RRMS and healthy controls [26]. The aim of the present study was to investigate whether LINE-1 methylation levels in circulating cfDNA are altered in RRMS. We have analyzed the methylation status of individual CpGs in LINE-1 repetitive elements in cfDNA isolated from serum of patients with RRMS and healthy subjects using bisulfite sequencing. The observed differences in LINE-1 cfDNA methylation were verified by quantitative PCR analysis of independent randomly selected samples of RRMS patients.

## Materials and Methods

### Patient Samples

The RRMS patient serum samples were collected at the Multiple Sclerosis Center (Nijmegen, The Netherlands) within the 2001–2015 time frame. Serum samples from healthy individuals were collected at the Sanquin Blood Bank (Nijmegen, The Netherlands) or at the Radboud University Medical Center (Nijmegen, The Netherlands). A cohort of 24 untreated RRMS patients in clinical remission was included in this study (Table 1); patients met the following inclusion criteria: (i) age greater than 18 years old; (ii) diagnosis of MS according to the McDonald criteria [27]. Patients diagnosed also with an autoimmune disorder other than MS were excluded from the study. Sera of 24 healthy individuals were used as controls (CTR; Table 1). Patient sera were collected in accordance with the code of conduct of research with human material in the Netherlands. Donors provided written informed consent. The serum samples were prepared according to standard protocols. After collection of the whole blood, the samples were left at room temperature for 1 h, and the clots were removed by centrifugation at 2000×*g* for 10 min. Sera were stored in aliquots at −80 °C.

**Table 1:** Characteristics of the patients with relapsing remitting multiple sclerosis and healthy subjects

Characteristics	CTR samples used forsequencing analysis (*n* = 6)	CTR samples used for qPCR analysis (*n* = 18)	RRMS samples used for sequencing analysis (*n* = 6)	RRMS samples used for qPCR analysis (*n* = 18)
Gender (male/female)	1/5	4/14	3/3	4/14
Age (years)	49.5 ± 6.0	47.5 ± 13.2	45.5 ± 5.4	43.5 ± 7.6
EDSS	NA	NA	2.8 ± 1.3	2.5 ± 1.7
Disease onset (years)	NA	NA	37.0 ± 4.3	33.5 ± 9.0

*EDSS* expanded disability status scale, *NA* not applicable

### DNA Isolation and Bisulfite Treatment

Circulating cfDNA was extracted from 200 μl serum using QIAamp®DNA blood mini kit (Qiagen, Hilden, Germany) according the manufacturer’s protocol. The isolated DNA was modified by sodium bisulfite treatment using the EpiTect Bisulfite kit (Qiagen, Hilden, Germany) according to the manufacturer’s instructions.

### LINE-1 Repeat Amplification, Cloning, and Sequencing

Six samples for each group were randomly selected for “bisulfite sequencing”. Bisulfite-treated DNA served as template for PCR-mediated amplification of a 376-bp amplicon using a set of primers (L1F/LR; Table 2) specific for bisulfite converted DNA [28]. High Fidelity Taq Polymerase (Roche, Basel, Switzerland) was used under the following conditions: 95 °C for 10 min, followed by 30 cycles at 95 °C for 90 s, 43 °C for 60 s, and 72 °C for 120 s. The PCR products were separated by 2% agarose gel electrophoresis, purified using QIAEX II Gel Extraction Kit (Qiagen, Hilden, Germany), cloned into the pCR4 TOPO vector (TA cloning kit, Invitrogen, Carlsbad, CA, USA) and sequenced. For each group 36 clones (6 per sample) were analyzed. We pooled the data from 36 sequences for each group to increase statistical power.

**Table 2:** Primers used for bisulfite sequencing and qPCR analysis

CpG	FORWARD PRIMERS (5′-3′)	REVERSE PRIMERS (5′-3′)
L1F1	AGTTAAAGAAAGGGGTGA	
L10	GTTAAAGAAAGGGGTGA**CG**GA**C**	
L18	GTATTAGGAGATTATATTT**C**	
L24	GAGATTAAATTGAAGGCGGTAA**CG**	
L27		AACCTAAACAATAA**CG**AA**CG**G
L1F	ATTTTATTAGGGAGTGTTAGATAGTG	
LR		AACTACTTTATTTACCCAAAC

The CpG positions are indicated for L1PA2 subfamily. The *bold letters* indicate CpG positions. L1F/LR set was used for sequencing. The primer combination was L10/LR for L1PA2-10, L18/LR for L1PA2-18, L24/LR for L1PA2-24, and L1F1/L27 for L1PA2-27, respectively

### Methylation-Specific Quantitative PCR

A methylation-specific quantitative PCR (qPCR) assay was used to detect methylation of LINE-1 CpG sites in cfDNA. The primers were designed to specifically bind to bisulfite-treated DNA. The primers specific for each CpG were designed based on the sequencing data in such a way that the discriminating nucleotide was positioned at or near the 3′ end of the primer. The sequences of the primers used are listed in Table 2. First, a LINE-1 fragment was amplified with the CpG-free L1F/LR primer set (35 cycles). This fragment was used as a template for qPCR analyses on a StepOnePlus qPCR machine (software version 2.2; Applied Biosystems, UK). qPCR reactions were performed in triplicate. Reactions were carried out in a final volume of 10 μl containing GoTag qPCR Master Mix (Promega, Fitchburg, Wisconsin, USA), 0.75 μM of each primer, 0.1 μl CXR reference dye, and 1 μl template DNA. The qPCR conditions were as follows: 95 °C for 10 min, followed by 40 cycles of 95 °C for 15 s, and 58 °C for 60 s. Unmethylated and fully methylated bisulfite-treated control DNA samples (EpiTect Control DNA, Qiagen, Hilden, Germany) were used as negative (0% methylated) and positive (100% methylated) controls, respectively. To measure the amount of total DNA and DNA methylated at the particular CpG site in each sample and amplification efficiency, standard curves were created by plotting the quantities of serially 10-fold diluted control 100% methylated DNA (Qiagen, Hilden, Germany), logarithmically against the Ct values. The primer set L1F/LR, which was designed to the LINE-1 areas free of CpG sites, has been used as internal control for normalization of DNA input (total DNA). Each primer set specific for a certain CpG site yields information on the amount of DNA methylated at the particular CpG site (quantity of methylated DNA). The primer combination was L10/LR for L1PA2-10, L18/LR for L1PA2-18, L24/LR for L1PA2-24, and L1F1/L27 for L1PA2-27, respectively. The relative methylation level for each “set of primers” was calculated according to the following formula: percentage of methylated DNA = (quantity of methylated DNA /quantity of total DNA) × 100.

### Data Analysis

High-scoring segment pair (HSP) distribution on genome was performed with the BLAT tools [29]. To quantify and compare the percentage of methylated CpG in both groups, the quantification tool for methylation analysis (QUMA) software was used [30]. In order to accurately determine the percentage of methylated LINE-1 using the qPCR assay, two different analysis methods were applied: a comparative quantification method based on quantification cycle (Cq) and the standard curve (SC), and the LinRegPCR method [31]. The comparative method relies on the assumption that the PCR efficiency is constant for the target and the reference amplicons. However, it has been shown that PCR efficiencies for target and reference amplicons often vary and this difference can lead to under- or overestimation of the target quantity. The LinRegPCR method allows the calculation of starting material and PCR efficiency for each individual sample.

A standard curve was generated by performing qPCR with a serial dilution of fully methylated DNA and was used to calculate the concentration of the cfDNA in the samples. The *C*_t_ values were plotted versus the log of the dilution. The efficiency was calculated based on the slope of the standard curve. The LinRegPCR method calculates efficiencies for each individual sample and uses the mean PCR efficiency per amplicon and the *C*_t_ value per sample to calculate the starting concentration per sample.

### Statistical Analysis

Statistical analysis was performed using SPSS 21.0 software (IBM SPSS Statistics for Windows, Version 21.0, Armonk, NY, USA). QUMA software performs a statistical analysis between the methylation profiles. Fisher’s exact test and the Mann-Whitney *U* test were used to determine the statistical significance of the difference for two groups at each CpG site or for the entire sets of CpG sites. All *p* values shown are for two-tailed tests with *p* < 0.05 considered significant.

## Results

### Patients and Controls

Two groups, each consisting of 24 subjects, were included in this study, RRMS patients in clinical remission and healthy controls (CTR). Characteristics of the study participants are summarized in Table 1. There were no significant differences in age and gender between the groups. The average age for the CTR and RRMS was 49 ± 14 and 46 ± 7 years, respectively, and the percentage of female subjects in both groups was high (85 versus 92%, respectively). Circulating cfDNA was isolated from serum samples of each of these subjects.

### Methylation Analysis of LINE-1 cfDNA Using Bisulfite Sequencing

**Fig. 1:**
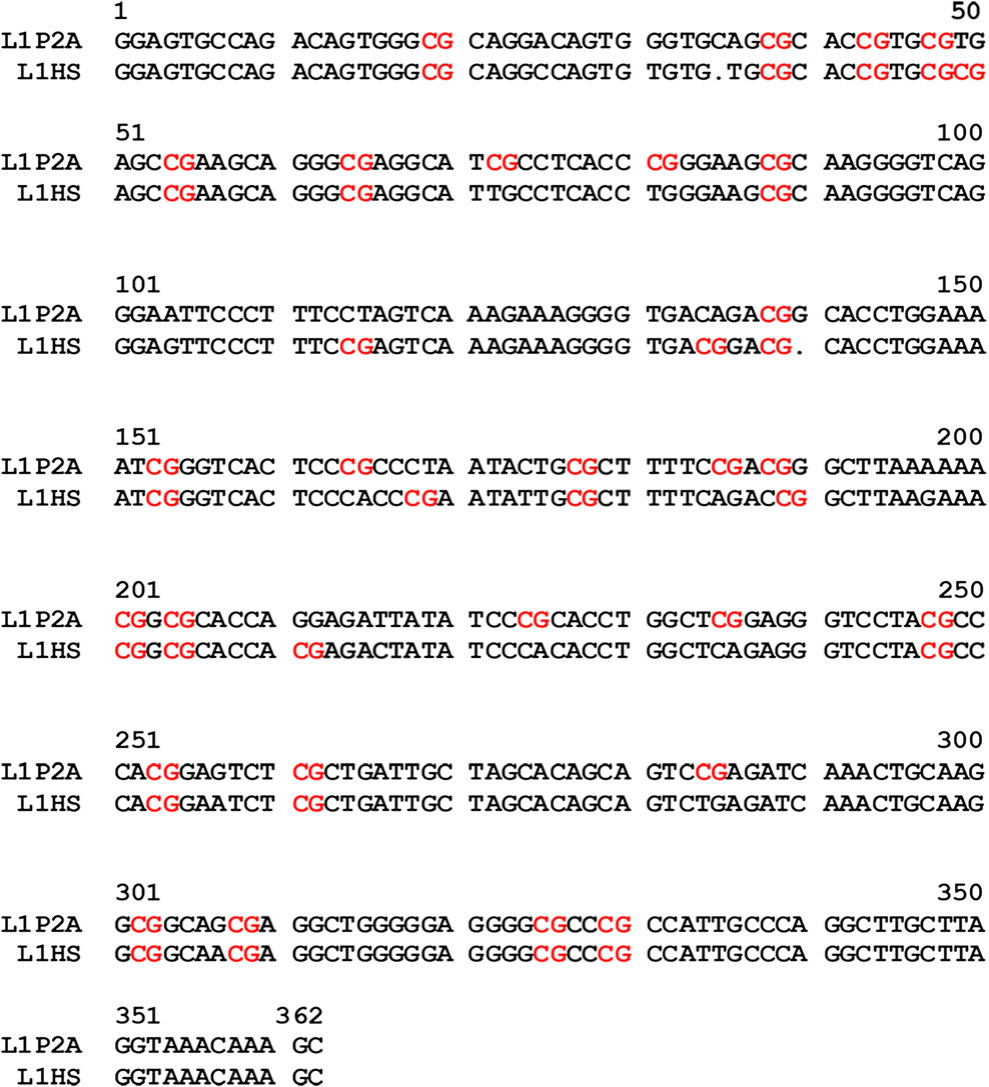
Sequence alignment of CpG islands of the L1PA2 and L1HS subfamilies. The positions of each CpG site are indicated in *red*. The L1PA2 and L1HS subfamily contain 27 and 25 CpG sites, respectively

To obtain information on the methylation state of individual CpG sites in the 5’UTR of LINE-1 in circulating cfDNA, the corresponding fragments were amplified by PCR (L1F/LR primer set) and cloned in the pCR4-TOPO vector. Thirty-six clones derived from 6 randomly selected RRMS subjects and 36 clones derived from 6 healthy subjects were sequenced (six clones per subject). In view of the copy number of full-length LINE-1 elements in the human genome, most, if not all, of these clones were expected to be derived from distinct LINE-1 elements. With the help of the software tool CENSOR [32], which screens a reference collection of repeats with a query, three LINE-1 subfamilies, L1PA2, L1Pt-5 end, and L1HS (human-specific L1, currently active gene in human genome) were identified. Of the CTR and RRMS sequences 41% and 32%, respectively, corresponded to L1HS, while the majority of the remaining sequences represented L1PA2 elements. Only two L1Pt-5 elements were found, both in CTR samples. The obtained sequences for L1PA2 and L1HS differed at several CpG positions (Fig. 1).

Methylation analysis was performed using the QUMA software. L1PA2 and L1HS showed the presence of 27 and 25 CpG sites, respectively (Fig. 1), 19 of which are shared by both subfamilies. The mean methylation level for the combined 27 sites of RRMS L1PA2 (50 ± 18%) was higher than that of CTR L1PA2 (40 ± 22%; *p* = 0.019). No differences between the mean methylation level for the combined 25 sites of RRMS L1HS (84 ± 11%) and CTR L1HS (84 ± 15%; *p* = 0.41) were observed.

**Fig. 2:**
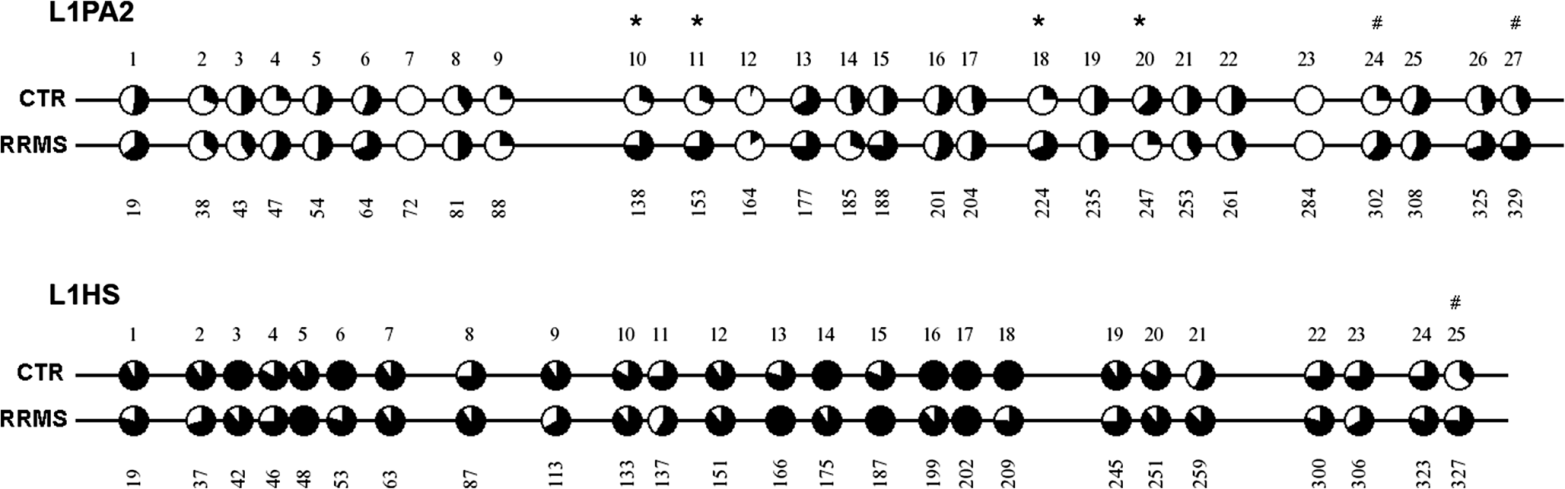
L1PA2 and L1HS methylation status in cfDNA determined by bisulfite sequencing. cfDNA was isolated from the serum of 6 healthy controls and 6 RRMS patients. After bisulfite treatment and cloning of the resulting DNA, 6 clones for each individual were selected and subjected to DNA sequence analysis. The methylation analysis was performed by Quantification Tool for Methylation Analysis (QUMA). The *schemes* show the methylation levels as pie charts for each individual CpG site in the L1PA2 subfamily and the L1HS subfamily. *Open circles* represent completely unmethylated sites and *filled circles* represent full methylation. The methylation level of each individual CpG site in RRMS and CTR was compared using Fischer’s exact test. *p < 0.05., CpG sites show a trend for higher methylation levels but the differences do not reach significance ^#^ *p* = 0.08, *p* = 0.09, and *p* = 0.08, respectively. The *numbers below* each CpG site indicate the position of the respective CpG site in the L1PA2/L1HS element

We next examined the methylation level at individual LINE-1 CpG sites in cfDNA. The methylation level varied considerably among individual CpG sites, ranging from 0 to 67% for CTR and from 0 to 78% for RRMS L1PA2 and ranging from 36 to 100% for CTR and from 60 to 100% for RRMS L1HS (Fig. 2). Three individual CpG sites (L1PA2 sites 10, 11, and 18) showed significantly higher methylation levels in the RRMS group compared with CTR (*p* < 0.05, Fig. 2). L1PA2 CpG site 20 displayed significantly lower methylation levels in RRMS compared with CTR (*p* = 0.04). CpG sites 24 and 27 in L1PA2 and CpG site 25 in L1HS showed higher methylation levels in RRMS patients compared to CTR, but the differences did not reach significance (*p* = 0.08 and *p* = 0.09, and *p* = 0.09, respectively). No significant differences between CTR and RRMS were observed for other individual CpG sites of L1HS (Fig. 2).

### Methylation Analysis of Individual LINE-1 CpG Sites by qPCR

To verify the methylation status of individual CpG sites, which were selected based upon the data described above, a CpG methylation-specific qPCR assay was developed and used to analyze cfDNA from distinct RRMS patients (*n* = 18) and control subjects (*n* = 18). These analyses were focused on L1PA2 CpG sites 10, 18, 24, and 27. Primer set L1F/LR, which is complementary to LINE-1 areas free of CpG sites, was used as internal reference for normalization of DNA input. The primers specific for each CpG site studied were designed based on the sequencing data in such a way that the discriminating nucleotide was positioned at or near the 3′ end of the primer. It is well known that mismatches located in the 3′ end region of the primer are detrimental for PCR amplification and have significantly larger effects on priming efficiency than more 5′ located mismatches [33–35].

**Fig. 3:**
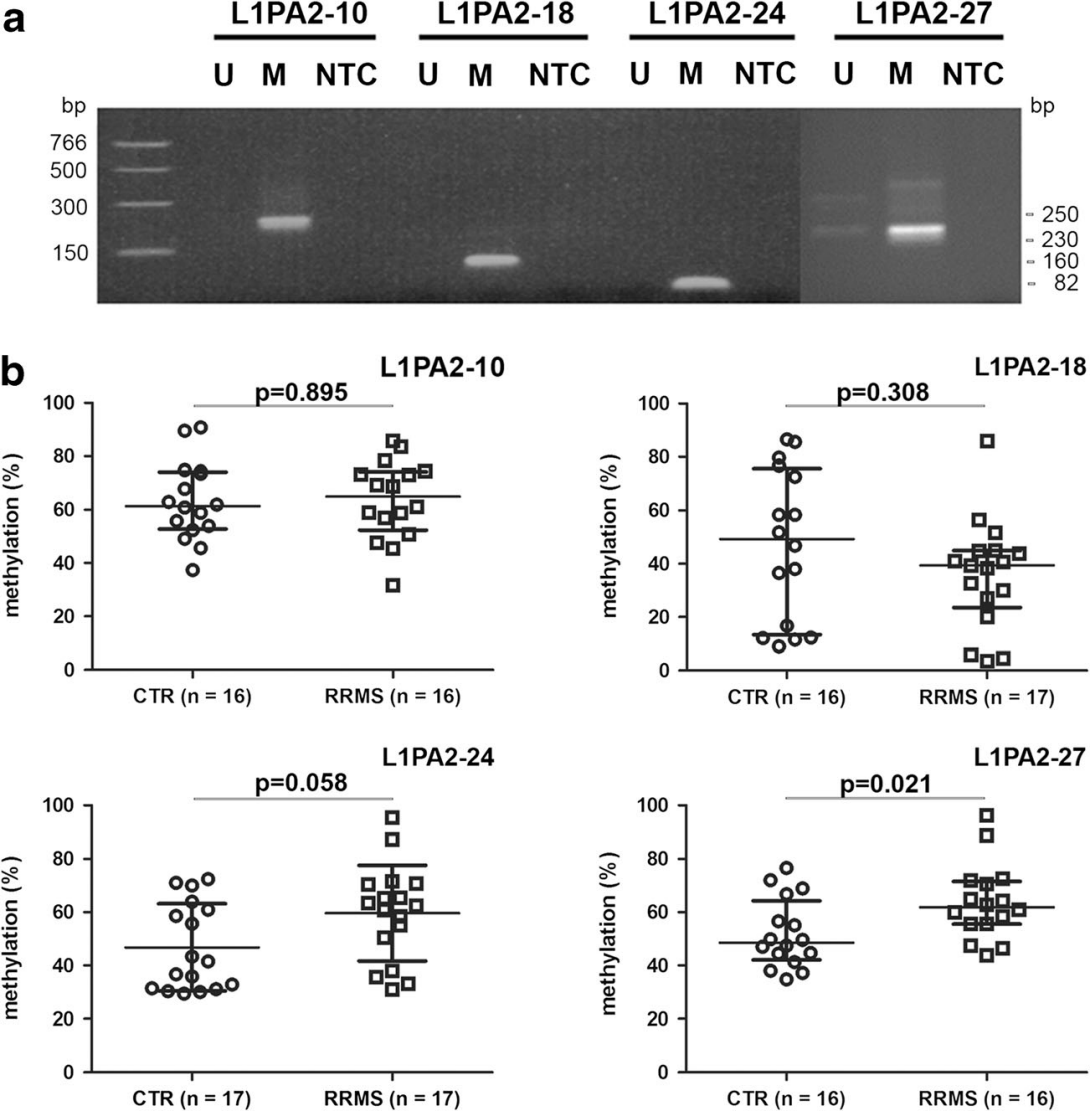
LINE-1 promoter CpG site analysis by methylation-specific quantitative PCR assay. **a** Analysis of PCR products by agarose gel electrophoresis to check primer specificity. *U* completely unmethylated DNA; *M* fully methylated DNA, *NTC* no template control, DNA size markers (base pairs) are shown on the *left*. The size (base pairs) of the amplicons is indicated on the *right*. **b** Methylation level for L1PA2 CpG sites 10, 18, 24, and 27 determined by qPCR using samples from RRMS patients and CTR. The *horizontal bars* indicate the median with interquartile range. The two groups were compared using unpaired Mann-Whitney tests

The specificity of the methylation-specific primers was established by PCR using either fully methylated or unmethylated bisulfite-treated DNA as positive and negative controls, respectively. These primers indeed resulted in PCR products only when methylated DNA was used as a template (Fig. 3a), confirming their specificity.

Both SC and LinRegPCR methods were applied to evaluate the methylation levels of CpG sites. The qPCR results revealed that only CpG site 27 showed significant difference in methylation levels between the RRMS and CTR groups (*p* = 0.02, Fig. 3b). There was a trend for higher methylation levels for CpG site 24, but it did not reach significance (*p* = 0.06). There was no significant difference in methylation levels for the other CpG sites (10 and 18) between RRMS and CTR. Thus, the qPCR results confirmed hypermethylation at L1PA2 site 27 in RRMS, as suggested by bisulfite sequencing. Multivariate analysis of variance (MANOVA) used to study the relationship between the methylation status of the L1PA2-24 and L1PA2-27 sites and RRMS demonstrated significantly higher methylation levels of these two sites in RRMS in comparison with CTR (*p* = 0.02). Thus, the methylation level of two CpG sites assayed in combination might increase the sensitivity of the assay. In addition, statistical analysis has shown that methylation levels of L1PA2 CpG sites 10, 18, 24, and 27 were not correlated with age, gender, and disease duration. No correlation was observed between methylation levels of L1PA2 CpG sites 18, 24, and 27 and Expanded Disability Status Scale (EDSS). The methylation level of L1PA2 CpG site 10 was significantly and negatively correlated with the EDSS score (the correlation coefficient is −0.69, *p* = 0.004).

## Discussion

In the present study, we analyzed the methylation pattern of the promoter region of LINE-1 repetitive elements in serum cfDNA from RRMS patients and healthy individuals using bisulfite sequencing and qPCR analysis. Two major LINE-1 subfamilies were identified in both groups, L1PA2 and L1HS. The L1PA2 subfamily represents an ancestral lineage and was found in the human and chimpanzee genomes [36]. The L1HS subfamily comprises relatively “young” LINE-1 elements and is specific for humans. Overall CpG methylation levels of L1PA2 subfamily fragments in cfDNA were significantly higher in RRMS than in CTR (50 ± 18 vs 40 ± 22%; *p* = 0.019). Higher L1PA2 methylation levels might be associated with lower expression levels. It has been shown that single or combinations of nucleotide differences within the LINE-1 5’UTRs influence the promoter activity and as a consequence transcriptional activity [37].

The L1HS fragments displayed higher overall methylation levels than L1PA2 fragments. No significant differences between the mean methylation levels of RRMS L1HS and CTR L1HS were observed (84 ± 11 vs 84 ± 15%; *p* = 0.41). The high overall L1HS CpG methylation levels in both groups are in agreement with the results of previously published studies, which demonstrated that young retrotransposon elements are more heavily methylated than more ancient elements, most likely to prevent their retrotransposition within the genome [9, 38]. Moreover, older elements also display more mutations due to deamination and/or nucleotide substitutions. Mutations within the LINE-1 promoter have the potential to reduce LINE-1 retrotransposition activity [39]. Indeed, we observed higher antisense deamination and mutation rates for the L1PA2 subfamily in comparison with the L1HS subfamily. No significant differences in antisense deamination and mutation frequencies were detected between control individuals and RRMS (data not shown).

We used two methods, bisulfite sequencing and qPCR with methylation-specific primers, to measure the methylation levels of individual CpG sites of LINE-1 repeats. Bisulfite sequencing revealed that the methylation levels varied considerably among individual CpG sites of LINE-1 elements and the methylation levels of several CpG sites differed between CTR and RRMS. L1PA2 CpG sites 10, 11, 18, 20, 24, and 27 showed 1.3–3.3-fold alterations in methylation levels. However, the analysis of a larger group of samples by qPCR demonstrated a significant difference only for CpG site 27. The lack of significance for CpG sites 10, 18, and 24 might be attributed to the small sample size used for bisulfite sequencing. Moreover, the high degree of homology between L1PA2 and L1HS primer sets for CpG sites 10 and 24 (11 and 22, respectively, in L1HS) most likely does not allow differentiation between the L1PA2 and L1HS sequences, and as a consequence, the qPCR results obtained for these sites have to be interpreted with care. In addition, due to the close proximity of some CpG sites, a few of these, which did not show any difference in methylation level, were present in the primers. These factors imply that qPCR analysis may be associated with an underestimation of methylation levels at sites 10 and 24.

Our results agree with those of recent studies on *T* cells showing that methylation of only a few CpG sites can be used to discriminate RRMS patients from CTR. Two CpG sites were hypermethylated in CD4+ and CD8+ *T* cells from MS patients compared to controls; one CpG upstream of the TMEM48 gene and another CpG in the last exon of the APC2 gene [40]. Another example of selective CpG site methylation differences was provided by studies of three CpG sites in blood samples, allowing reliable age prediction [41, 42]. Moreover, hypermethylation of CpG sites in repetitive elements (Alu, LINE-1, and SAT-a) in whole blood from MS patients compared to healthy controls was observed [25]. Thus, methylation patterns of cfDNA might reflect the methylation changes in blood cells, which is consistent with the fact that cfDNA originates from these cells.

One would expect to find a relatively large variety of circulating DNA methylation patterns in different individuals due to possible variations in cell number, cellular heterogeneity, age, gender, enzymatic activities and technical variations in sample preparation. In this respect, it is also important to note that it has been demonstrated that methylation levels of LINE-1 elements from different loci can be different [16]. All these factors could confound the LINE-1 methylation analysis. However, the results of the current study strongly suggest that serum cfDNA methylation might serve as a reliable surrogate marker for multiple sclerosis, provided the analyses are performed in a systematic manner. To explore its applicability, samples from larger and independently collected cohorts, and cohorts of other subtypes of MS, need to be analyzed. In addition, data on samples from early MS and pre-disease patients will be required to assess its predictive value.

The assessment of global DNA methylation is often performed via the analysis of the methylation status of repetitive elements [43–46]. However, in general the number of CpG sites assessed is rather small (2 to 4 CpG sites), especially when methylation is analyzed by restriction enzyme analysis (combined bisulfite restriction analysis; COBRA). Our results indicate that the results of LINE-1 CpG methylation to assess global methylation should be interpreted with care due to the relatively large differences in the methylation levels of individual sites.

In summary, the results of this study indicate that the analysis of overall LINE-1 methylation levels in serum cfDNA to discriminate RRMS patients from CTR is feasible for the L1PA2 subfamily. In addition, our results suggest that the methylation status of specific CpG sites may provide a basis for a molecular marker for RRMS: a significant increase in methylation of L1PA2 CpG site 27 was observed in circulating cfDNA of RRMS patients. Thus, the methylation status of circulating cfDNA may reflect pathophysiological phenomena in the brain. More extensive studies are needed to further characterize the association of the methylation status of cfDNA and multiple sclerosis.

## Electronic supplementary material

Figure S1 Schematic diagram of full length of LINE-1 repeats**.** Human LINE-1 consists of four main domains: 5’and 3’UTR untranslated regions and two open reading frames (ORFs), which encode two proteins required for retrotransposition. The promoter region containing the CpG sites is indicated. LINE-1 transcripts terminate with a poly(A) tail. SP and ASP, sense and antisense promoters, respectively. (GIF 4 kb)

High resolution image (TIFF 1035 kb)
