# Supplementary figures and images for: LINE-1 Hypermethylation in Serum Cell-Free DNA of Relapsing Remitting Multiple Sclerosis Patients
Source: Mol Neurobiol. 2017 Jul 13;55(6):4681–8. doi: 10.1007/s12035-017-0679-z (PMC5948235; doi:10.1007/s12035-017-0679-z)

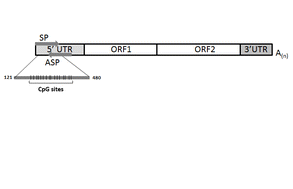

Supplement: Supplementary file 1 — Schematic diagram of full length of LINE-1 repeats. Human LINE-1 consists of four main domains: 5’and 3’UTR untranslated regions and two open reading frames (ORFs), which encode two proteins required for retrotransposition. The promoter region containing the CpG sites is indicated. LINE-1 transcripts terminate with a poly(A) tail. SP and ASP, sense and antisense promoters, respectively. (GIF 4 kb) [file 12035_2017_679_Fig4_ESM.gif]

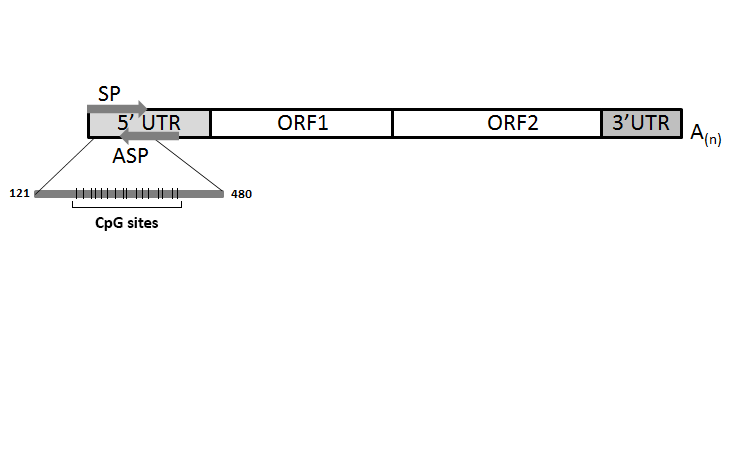

Supplement: Supplementary file 2 — High resolution image (TIFF 1035 kb) [file 12035_2017_679_MOESM1_ESM.tif]
